# Perforin Rapidly Induces Plasma Membrane Phospholipid Flip-Flop

**DOI:** 10.1371/journal.pone.0024286

**Published:** 2011-09-12

**Authors:** Sunil S. Metkar, Baikun Wang, Elena Catalan, Gregor Anderluh, Robert J. C. Gilbert, Julian Pardo, Christopher J. Froelich

**Affiliations:** 1 Department of Medicine, NorthShore University HealthSystems Research Institute, Evanston, Illinois, United States of America; 2 Department of Biology, Biotechnical Faculty, University of Ljubljana, Ljubljana, Slovenia; 3 Division of Structural Biology, Henry Wellcome Building for Genomic Medicine, Oxford, United Kingdom; 4 Departamento Bioquimica y Biologia Molecular y Cellular, University of Zaragoza, Zaragoza, Spain; 5 Fundación Aragón I+D, Zaragoza, Spain; Consejo Superior de Investigaciones Cientificas, Spain

## Abstract

The cytotoxic cell granule secretory pathway is essential for host defense. This pathway is fundamentally a form of intracellular protein delivery where granule proteases (granzymes) from cytotoxic lymphocytes are thought to diffuse through barrel stave pores generated in the plasma membrane of the target cell by the pore forming protein perforin (PFN) and mediate apoptotic as well as additional biological effects. While recent electron microscopy and structural analyses indicate that recombinant PFN oligomerizes to form pores containing 20 monomers (20 nm) when applied to liposomal membranes, these pores are not observed by propidium iodide uptake in target cells. Instead, concentrations of human PFN that encourage granzyme-mediated apoptosis are associated with pore structures that unexpectedly favor phosphatidylserine flip-flop measured by Annexin-V and Lactadherin. Efforts that reduce PFN mediated Ca influx in targets did not reduce Annexin-V reactivity. Antigen specific mouse CD8 cells initiate a similar rapid flip-flop in target cells. A lipid that augments plasma membrane curvature as well as cholesterol depletion in target cells enhance flip-flop. Annexin-V staining highly correlated with apoptosis after Granzyme B (GzmB) treatment. We propose the structures that PFN oligomers form in the membrane bilayer may include arcs previously observed by electron microscopy and that these unusual structures represent an incomplete mixture of plasma membrane lipid and PFN oligomers that may act as a flexible gateway for GzmB to translocate across the bilayer to the cytosolic leaflet of target cells.

## Introduction

The granule secretory pathway represents an important host defense against tumor and pathogen infected cells. This pathway is fundamentally a form of intracellular protein delivery where the pore forming protein perforin (PFN) contributes to the delivery of the granule proteases (granzymes), which in turn then mediate cytotoxic as well as additional biological effects. Although PFN and granzymes were first discovered more than 25 years ago [1], [2], [3], the mechanism through which PFN remodels the target cell plasma membrane for granzyme passage across the bilayer remains elusive. The original model proposed that the proteases simply diffuse through barrel stave pores generated in the plasma membrane of the target cell [4]. Recent structural studies have provided images indicating that this pore consists of a ring of about twenty subunits with a diameter of approximately 20 nm [5].

Using electron microscopy or other biophysical approaches, pores of various functional diameters are observed on membranes when PFN is added as the isolated protein or via cytotoxic cells [1], [6], [7], [8], [9], [10], [11], [12]. The direct observation of the movement of cationic proteases across the plasma membrane of target cells through such pores remains unachieved and, perplexingly, granzyme delivery seems to occur without detectable pore formation [13], [14], [15]. A fundamental consideration in evaluating this paradox is to ask what form PFN monomers adopt to effect protein delivery. For example, what is the relevant number of PFN molecules that a target cell needs to encounter to achieve this goal? Experimentally, sufficient quantities of PFN, either in isolated form or secreted by a cytotoxic cell, will readily induce target cell necrosis while much lower concentrations, which leave the membrane apparently unscathed, are necessary to deliver the granzymes.

An alternative model proposes that PFN and granzymes are autonomously internalized within endocytic vesicles from which delivery occurs by PFN-mediated endosomolysis [16]. Another variant proposes that PFN pores generate sufficient calcium influx to trigger a membrane repair response which drives internalization of the granzyme for subsequent endocytic delivery [17]. We have attempted to visualize endosome lysis caused by PFN using CLSM, without success (Metkar and Froelich, unpublished). It remains unclear therefore how granzymes are delivered across either the plasma or (if involved) the endosomal membrane. An additional problem is our inability to identify target cells prepared by PFN for granzyme delivery.

To evaluate the effect of PFN monomers on the plasma membrane of target cells, we have used probes that assess permeability as well as alterations in membrane composition. When administered alone, PFN, as determined by Annexin-V (Ann-V) staining, causes the target cell to externalize PS from the internal to external leaflet. The pattern of staining is distinct from that observed for necrotic and apoptotic cells and the presence of exposed PS is also detected by lactadherin (LA) binding. PS externalization was shown to occur in target cells even though such fluorophores as Propidium Iodide (PI) and Sytox Green (SG) are completely excluded in the presence of sufficient quantities of human perforin that deliver the granzyme. Importantly, PS externalization was also observed after antigen specific CTLs contacted peptide-pulsed target cells. PFN therefore appears to induce a rapid re-organization of the plasma membrane, namely PS flip-flop [18], [19]. A possible explanation for this phenomenon is the formation of short-lived, low-caliber pores that consist of a matrix of lipid and PFN oligomers. Related configurations have been described for the pro-apoptotic protein, Bax [20], for equinatoxin [21] and during membrane fusion [22], [23]. The formation of such structures might suggest a mechanism for traversal of granzymes across the cell boundary, gliding over the curved lipid surface. Furthermore, matrices of protein and lipid have increased flexibility compared to protein channels, which suggests an unanticipated mechanism for translocation of granzymes across a formidable barrier- the lipid bilayer.

## Results

### Isolated PFN is rapidly inactivated by extracellular Ca ions

Following cytotoxic cell degranulation, PFN monomers dissociate from their carrier, the proteolgycan, serglycin, and face two outcomes. Serum proteins [24] and, possibly Ca ions may inactivate PFN monomers while others undergo well studied, Ca-dependent binding to plasma membrane phospholipids [25], [26], [27]. We learned that pre-treating PFN with Ca results in a rapid decay in permeabilizing activity with more than 80% activity lost in approximately 5 min (Figure S1a). Furthermore, using EM to visualize the effect of Ca ions, PFN monomers were observed to assemble into ring-shaped oligomers in solution with an appearance comparable to the cylinders observed on membranes (Figure S1b). Similar to solution oligomerization that inactivates the related cholesterol-dependent cytolysin, pneumolysin [28], Ca induces the oligomerization of PFN, depleting the supply of monomer that is able to interact with the target cell membrane.

### Isolated PFN and GzmB initiates the lethal hit within minutes

**Figure 1:**
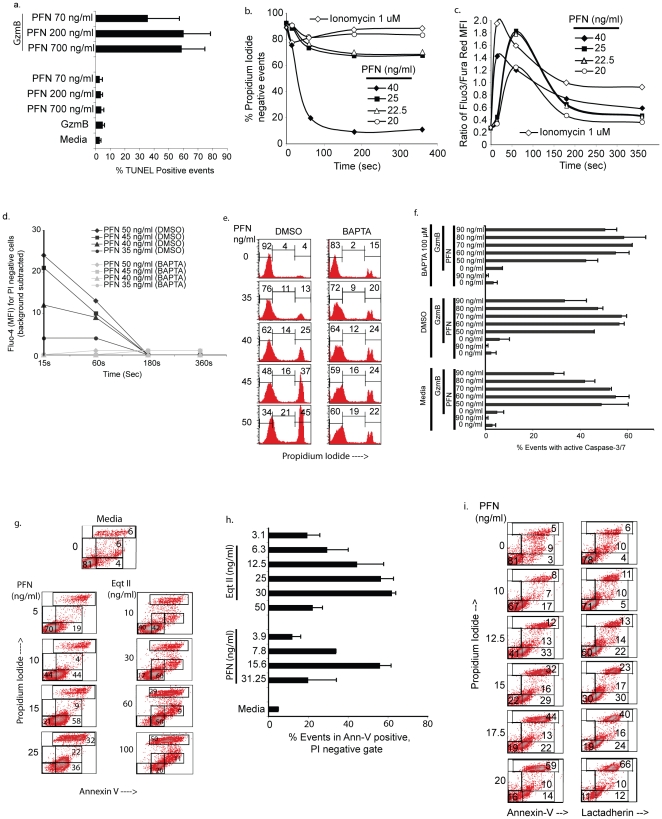
A brief pulse of target cells with human PFN induces calcium influx and causes PS flip-flop without evidence of membrane pore formation as measured by the cationic fluorophore propidium iodide. *a. Five min pulse with PFN and GzmB is sufficient to induce apoptosis (TUNEL).* Jurkat cells were pulsed with GzmB (1 µg/ml) and PFN at the indicated concentrations for 5 min. The reaction was then stopped by an EGTA wash step. Cells were incubated for 90 min after which they were fixed and stained for TUNEL reactivity (mean + sd, n = 3). *b and c. Calcium influx occurs in PFN-treated cells that exclude PI.* Fluo-3 and Fura Red loaded Jurkat cells were treated with indicated concentrations of PFN in the presence of PI and the percent events in PI negative subset enumerated over 6 minutes (b). Intracellular Calcium flux was measured concomitantly using the ratio of Fluo-3/Fura Red MFI in the PI negative subset (c). Cells were followed to 6 minutes. Data is representative of one of three independent experiments. *d. PFN mediated Ca influx can be blocked by BAPTA-AM.* Fluo-4 and BAPTA-AM/DMSO loaded cells were treated with the indicated concentrations of PFN in presence of PI and intracellular Calcium flux measured in PI negative cells. Data is presented as background subtracted Fluo-4 MFI over the indicated times and is representative of 1 of 2 independent experiments. *e. Intracellular Calcium chelation does not increase PFN induced necrosis.* BAPTA-AM/DMSO loaded cells were treated with the indicated concentrations of PFN in presence of PI for 6 min and analyzed by flow. Data is representative of 1 of 2 independent experiments. Numbers represent percent events in the various gates. f. *Inhibition of Ca influx does not interfere with PFN-mediated granzyme delivery.* Media, DMSO or BAPTA-AM loaded cells were treated with GzmB and PFN at the indicated concentrations for 1 hour at 37°C in presence of the Cell Event Caspase 3/7 reagent and PI. Cells demonstrating activated Caspase-3/7 were enumerated using flow cytometry (mean + sd, n = 2). *g. PS flip-flop occurs in PFN-treated cells that exclude PI – annexin-V binding.* Jurkat cells were treated with PFN for 15 min, washed and stained with Ann-V-FITC. PI was present throughout the assay. Eqt II was used as a positive control. Data from one representative experiment is shown. Numbers represent percent events in the various gates. *h. PFN induces PS flip-flop in cells that exclude PI.* Jurkat cells were treated as described above and events in the Ann-V positive, PI negative gate induced after PFN and Eqt II treatment enumerated for three experiments (mean + sd). *i. PS flip-flop occurs in PFN-treated cells that exclude PI –Lactadherin binding.* Jurkat cells were pulsed with indicated concentrations of PFN for 15 min in presence of PI, washed and stained with either Ann-FITC or Lactadherin-FITC in calcium containing buffer. Percent events in the respective gates are depicted.

Cytotoxicity assays that examine the potency of PFN and granzyme B (GzmB) *in vitro* involve an incubation step in which the proteins are mixed with target cells for four hours or longer and thereafter, a number of cell death parameters are measured. Since PFN will be largely inactivated within minutes by Ca, we made use of this insight to learn whether a brief exposure to PFN and GzmB was sufficient to induce target cell apoptosis. A 5 minute pulse with PFN and GzmB was quite sufficient to induce target cell apoptosis as shown by the TUNEL assay which measures DNA fragmentation (Fig. 1a)(see Figure S1c for alternate readout after a 15 min pulse and 1 hour incubation). These data suggest that the number of monomers successfully binding to the target membrane, and the time available to initiate a lethal hit, will be limited during *in vitro* assays.

### Further efforts to identify pores induced by sublytic PFN

The study of PFN-dependent granzyme delivery *in vitro* generally entails two steps. First, PFN is titrated against the target cells of interest to identify the percentage of permeabilized cells at various concentrations distinguishing the necrotic cells that develop. Minimally permeabilizing (sub-lytic) concentrations of PFN are then chosen for the delivery studies. The addition of PFN to target cells in the presence of PI yields two subsets: 1) undamaged target cells and 2) necrotic cells. The subset of interest, that is, target cells prepared for granzyme delivery, resides among the apparently normal population. As the concentration of PFN increases, the percentage of necrotic cells rises until all the cells die presumably due to the formation of enough pores to cause a massive Ca influx [29]. To our knowledge, markers that identify the PFN-induced membrane changes that might be associated with granzyme delivery have not been described. Indeed, we have been unable to identify pores in target cells exposed to concentrations of native human PFN that cause granzyme delivery but not necrosis using fluorophores such as Propidium Iodide (PI) [13] or Sytox Green (SG) (data not shown), even when the fluorophores are added to target cells *prior* to PFN. This issue has perplexed us because clearly 20 nm pores are observed when recombinant PFN is added to liposomal membranes [5] and, as determined by an osmotic protection assay, human PFN forms structures in erythrocytes that have a hydrodynamic diameter between 2.7 nm and 3.2 nm (Figure S1d). These values agree with a number of studies that examine the pore diameter produced by human and mouse PFN in erythrocytes [8], [9], [11].

Although fluorophores appear to be excluded from target cells that are exposed to concentrations of human PFN that cause granzyme delivery, calcium influx is quite evident (Fig. 1b, c) [17]. Thus, the measurement of Ca influx in otherwise normal-appearing PFN-treated cells may be a useful marker to define the subset that is rendered susceptible to granzyme translocation. In addition, our failure to identify pores with such fluorophores as PI may be due to the presence of the pore structures at low density on the target cells as well as due to their rapid clearance by membrane repair [17], [30]. To address these possibilities, we examined whether inhibition of Ca-mediated membrane repair with the chelator, BAPTA-AM slows Ca influx and thus reduces GzmB-induced apoptosis and reveals pores in target cells that are identifiable with PI. Here, the slowing of membrane repair should prolong the half-life of PFN pores in the plasma membrane of the target cells, allowing the entry of fluorophore and shifting the PFN concentration curve leading to more necrotic cells at lower concentrations of PFN [30]. The validity of this approach is described for SLO (see Figure S1e). Unlike the technique used to examine SLO treated cells which entails the examination of PI entry in the presence and absence of extracellular Ca ion, BAPTA-AM was used to effectively chelate the influx of Ca induced by a range of non-toxic concentrations of PFN (Fig. 1d). Nevertheless, an increase in PI uptake was not discernible (Fig. 1e) and, surprisingly, the inhibition of Ca influx did not reduce the induction of cell death (Fig. 1f). It thus seems that isolated human PFN generates structures sufficient for entry of Ca ions but not low molecular weight cationic fluorophores such PI and SG (600–700 daltons) and this signal does not contribute to granzyme delivery.

### PFN alone rapidly induces PS externalization

To investigate the membrane alterations allowing Ca entry we evaluated probes of membrane reorganization, such as the migration of inner leaflet-specific components (e.g. phosphatidylserine - PS) to the outer leaflet of the bilayer [19], [31]. Two well-known ligands of PS, the proteins, Annexin-V (Ann-V) and lactadherin [32] were examined. We found using 5 min (data not shown) or 15 minute pulses that human PFN rapidly induces PS externalization as detected with Ann-V binding, without allowing the passage of PI (Fig. 1g, see bottom right gate). Notably, Ann-V stains the PFN-treated cells at a lower intensity compared to the pattern observed for the discrete population of necrotic cells (top right gate). Equinatoxin II (Eqt II), a toxin predicted to generate pores from a matrix of protein and lipid [21], [33], produced a similar pattern of low-intensity Ann-V reactivity in nucleated cells (Fig. 1g, see bottom right gate). Fig. 1h summarizes these data for PFN and Eqt II. PFN induced PS externalization is also recognized by lactadherin, a probe that binds to the anionic lipid independent of calcium (Fig. 1i). The absence of PI influx makes it implausible that the 40–45 kDa Ann-V and lactadherin stain cells by entering and binding PS in the plasma membrane inner leaflet, and we therefore conclude that the probes must be binding PS that PFN, on a rapid timescale, has induced to migrate to the outer leaflet.

### PS externalization induced by PFN is not mediated by Ca influx

**Figure 2:**
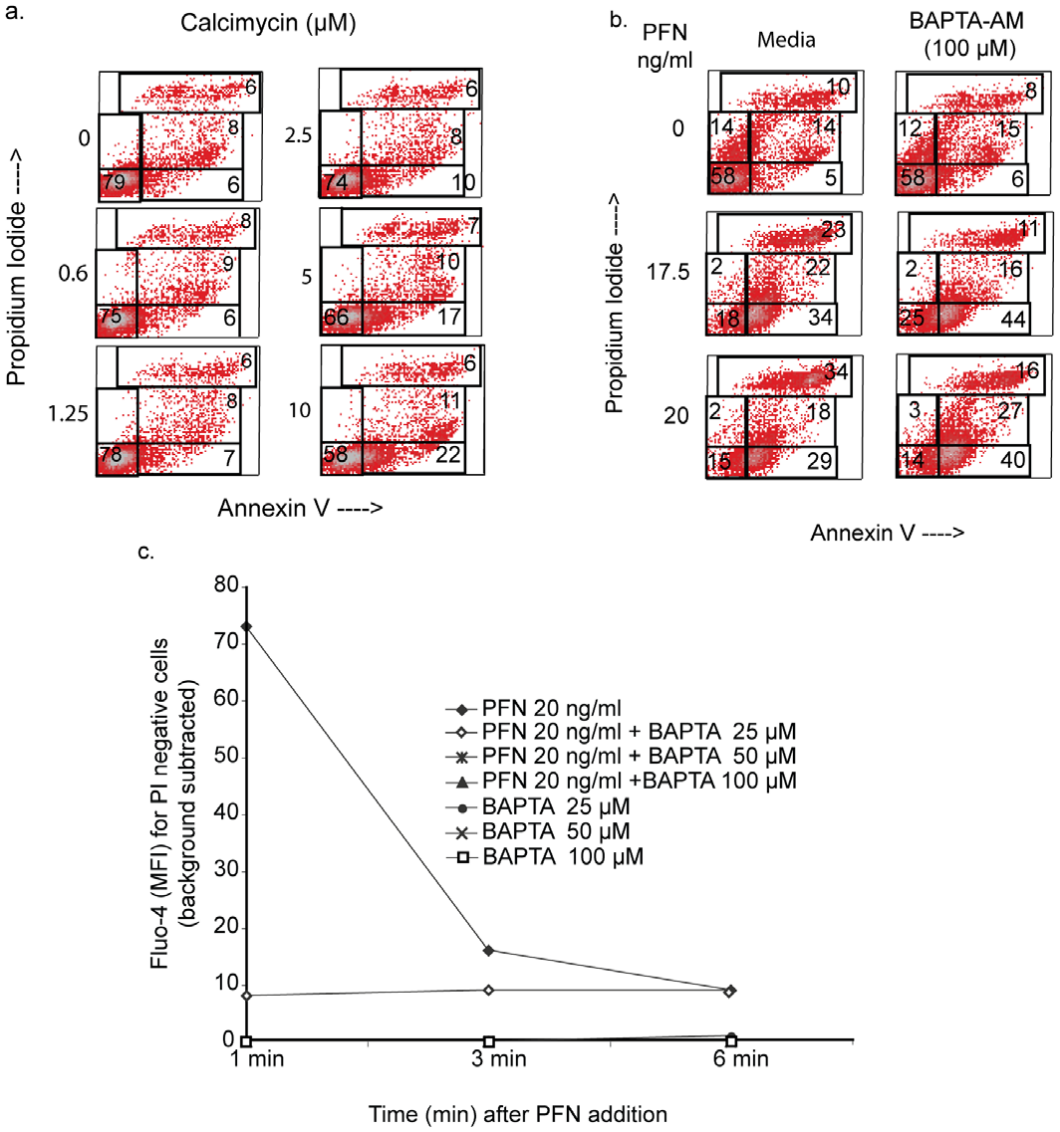
PFN-mediated Calcium influx does not induce Ann-V reactivity in Jurkat cells. *a. Calcimycin treated Jurkat cells demonstrate PS flip-flop.* Cells were exposed to Calcimycin at the indicated concentrations for 15 min in presence of PI, followed by Ann-V-FITC staining. Numbers represent percent events in the respective gates. Data described for one of two experiments. *b. Ann-V reactivity in PFN treated cells is not inhibited by calcium chelation.* Cells were loaded with BAPTA-AM, treated with human PFN in presence of PI and stained with AnnV-FITC. Density plots from 1 of 2 independent experiments is shown. c. *Intracellular Calcium chelator BAPTA-AM prevents Calcium flux in cells treated with PFN.* Jurkat cells were loaded with Fluo-4 with/without BAPTA-AM, washed and treated with PFN in presence of PI. Calcium flux was measured over 6 min. Data is presented as background subtracted Fluo-4 MFI over the indicated times and is representative of 1 of 2 independent experiments.

Next we asked whether Ann-V and lactadherin binding induced by limiting concentrations of PFN could be due to calcium influx and activation of PS translocases [34], [35]. A calcium ionophore did indeed induce measurable but minor Ann-V reactivity when applied to the Jurkat cells (Fig. 2a). However, when the effects of calcium influx were minimized by pre-loading targets with BAPTA-AM, human PFN still elicited a sizable Ann-V reactive subset (Fig. 2b–c).

### PS externalization induced by PFN is enhanced by phospholipids that increase membrane curvature and by cholesterol depletion of the plasma membrane

**Figure 3:**
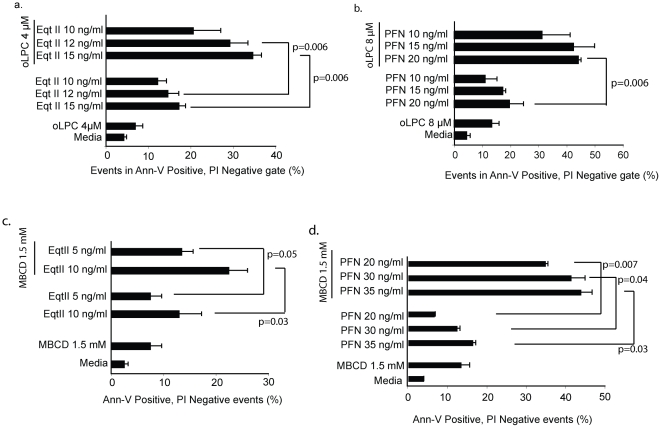
Effect of long chain positive curvature lipid, oLPC and Cholesterol depletion on Eqt II and PFN-induced PS flip-flop. *a. LysoPC augments Eqt II induced PS flip-flop.* Cells were treated with Eqt II in the presence and absence of oLPC for 15 min, washed and then stained with Ann-V-FITC. Data describe percent events in the Ann-V positive, PI negative gate (mean ± sd, n = 3). *b. LysoPC augments PFN induced PS flip-flop.* Cells were treated with oleoyl LysoPC for 15 min to allow for plasma membrane insertion, washed and then treated with PFN at the indicated concentrations for 15 min. Cells were washed and stained with Ann-V-FITC. PI was present throughout the assay. Data (mean ± sd) represent percent events in the Ann-V positive, PI negative gate for two independent experiments. c. *Cholesterol depletion augments Eqt II induced PS flip-flop.* Cells pre-treated with MBCD were treated with Eqt II for 15 min, washed and then stained with Ann-V-FITC. Data describe percent events in the Ann-V positive, PI negative gate (mean ± sd, n = 2). *d Cholesterol depletion augments PFN induced PS flip-flop.* Cells pre-treated with MBCD were exposed to PFN at the indicated concentrations for 15 min. Cells were washed and stained with Ann-V-FITC. PI was present throughout the assay. Data (mean ± sd, n = 2) represent percent events in the Ann-V positive, PI negative gate for two independent experiments. A two-tailed paired t-test was used to determine statistical significance.

Since PFN appears to be directly inducing reconfiguration of the plasma membrane lipids, we then sought to investigate the lipid dependence of this phenomenon. To do this we made use of oleoyl lysophosphatidylcholine (oLPC), a phospholipid that induces positive membrane curvature [31], [36], [37] and by reducing the cholesterol content of the plasma membrane. We first demonstrated the suitability of this approach by showing that the oLPC increased the percentage of Ann-V reactive cells among Eqt II treated target cells (Fig. 3a). This technique however was problematic for the study of PFN because the assay ordinarily is performed in the presence of fluid-phase oLPC, a condition that inhibits the capacity of the PFN monomers to bind to the plasma membrane of the target cells due to their interaction with the LPC headgroups [38]. Therefore, Jurkat cells were preincubated with oLPC, exposed to PFN and, then monitored for PS flip-flop. Despite the limited exposure, the data show that the pre-incubation of the target cells with the oLPC increased the percentage of Ann-V positive cells after the target cells were exposed to PFN (Fig. 3b).

Depleting the relatively rigid cholesterol molecule from the target cell plasma membrane mediated by non-toxic concentrations of methyl-β-cyclodextrin (MBCD) was also observed to increase PS flip-flop induced by both Eqt II and PFN (Fig. 3c–d). Among the many effects that cholesterol has on the organization of the plasma membrane, its removal is predicted to increase the disorder of the phospholipid acyl chains as well as the size of ordered and disordered domains [39], [40], [41]. These observations further strengthen the likelihood that PS flip-flop originates from reconfigurations among the lipids themselves by PFN.

### PFN-induced PS flip-flop is the first marker that correlates with a cell's susceptibility to GzmB-induced apoptosis

**Figure 4:**
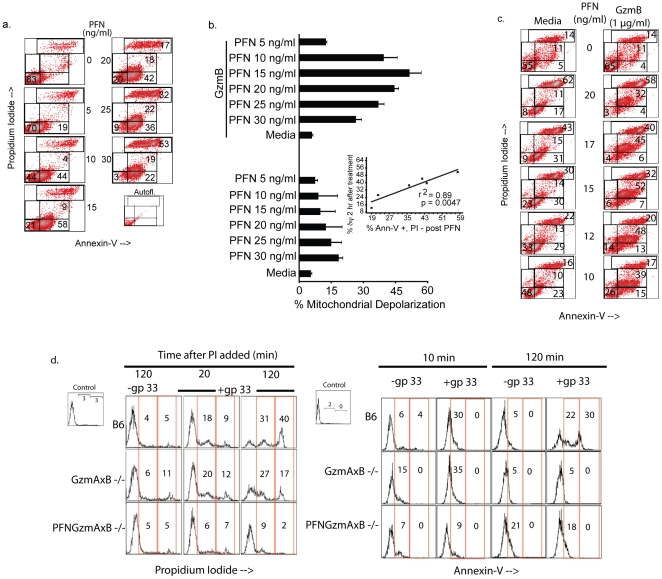
Cells demonstrating PS flip-flop are susceptible to GzmB induced apoptosis. *a. PFN induces PS flip-flop in PI negative cells.* Cells were treated with PFN, washed, and stained with Ann-V-FITC. GzmB (1 µg/ml) was added to paired samples to assess delivery (see Fig. 4b). An auto-fluorescence control is also shown. *b. The presence of Annexin V reactivity (PS flip-flop) correlates with susceptibility of target cells to GzmB-mediated apoptosis.* Paired sample (PFN alone and PFN plus GzmB) were incubated for 45 min in presence of CMX-ROS. PFN concentrations were chosen for their ability to induce PS flip-flop in PI negative cells from (a). Cells undergoing mitochondrial depolarization were enumerated and results are presented as mean + sd of 2 experiments. Inset: A significant correlation (r^2^ = 0.89; p = 0.0047) was observed between PI negative events exhibiting flip-flop in presence of PFN and events describing mitochondrial depolarization in presence of PFN and GzmB. *c. A comparison of PS flip-flop in PFN versus PFN-GzmB pulsed Jurkat cells.* To compare the pattern of Ann-V-FITC staining produced by PFN versus PFN plus GzmB, cells were pulsed with PFN in the presence and absence of GzmB (1 µg/ml) for 15 min at 37°C followed by Ann-V-FITC staining (data for one of two experiments). *d. gp33 specific murine CTL that contain PFN induce Ann-V + subset in gp33 loaded targets.* Ex vivo CD8 cells from LCMV mice were isolated, labeled with CellTracker Green and added to EL4 cells ± gp33 at E∶T of 2∶1. PI (10 µg/ml) was added at onset with distribution of PS flip-flop and PI reactivity within targets determined by FACS at 10, 20 and 120 min in the CellTracker negative population (EL4 cells). Data representative of 1 of 2 independent experiments.

Since the measurement of Ca influx was not a reliable marker for granzyme delivery, we examined whether PFN-induced PS flip-flop correlated with cellular susceptibility to GzmB. Targets were split into two aliquots with one exposed to PFN alone to measure Ann-V and PI reactivity (Fig. 4a) while the other was treated with both PFN and GzmB followed by measurement of cell death by mitochondrial depolarization (Fig. 4b). The results show a statistically significant correlation between the identified Ann-V reactivity and the percentage of cells that undergo apoptosis (r^2^ = 0.89, p = 0.0047; Fig. 4b inset). These data therefore suggest that the low intensity Ann-V positive subset induced by PFN identifies a portion of the cells that undergo apoptosis if exposed to GzmB. For comparison, the pattern of Ann-V staining is presented after a 15 min PFN-GzmB pulse (Fig. 4c) as well as after a 2 hr incubation period to verify that the pulse resulted in apoptosis (Figure S1f). GzmB, despite the short exposure time, modified the pattern generated by PFN producing a low intensity PI population as well as the low intensity Ann-V cells, cells.

### PFN-induced PS flip-flop is induced by antigen specific CTLs

**Figure 5:**
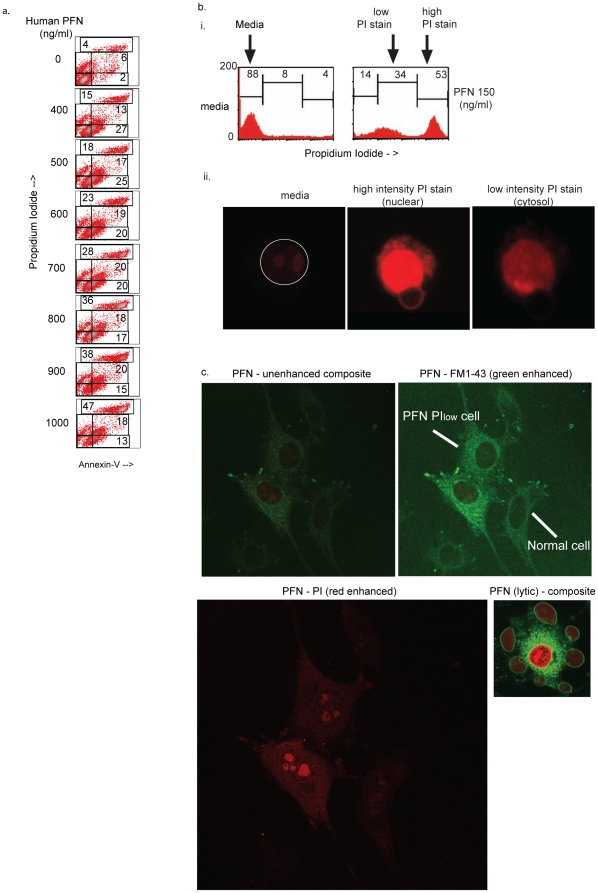
PFN induces both low intensity PI and Ann-V reactivity in target cells. *a. Target cells treated with human PFN under low calcium develop both low intensity PI and Ann-V reactivity.* Jurkat cells were treated with human PFN in the presence of 0.2 mM calcium, washed with 0.2 mM calcium containing buffer and then stained with Ann-V-FITC in presence of 1.25 mM calcium. Density plots from 1 of 2 independent experiments is shown. *b. A PI low intensity subset is induced by PFN as visualized by flow cytometry and CLSM:* Jurkat cells were treated with rat PFN in the presence of PI for 15 min, washed and either run on a flow cytometer (i) or imaged by CLSM (ii) as describe in methods. Histograms show PI staining where normal, low intensity and high intensity PI subsets are marked along with the percentages in the respective regions. Confocal images visualizing the PI hi and low cells along with Media control are shown in the lower panel. Data is representative of 1 of 3 experiments. **c.** *Imaging of low intensity PI cells induced by PFN:* HeLa cells were treated with PFN (150 ng/ml) in the presence of FM1-43 and PI and imaged by CLSM. The upper left panel shows unenhanced image of three Hela cells, two with low intensity PI uptake and one unaffected (normal). The upper right panel show the same field with green channel (FM1-43) enhancement. Enlarged lower left panel show enhancement of red channel to visualize PI. Lower right panel depicts PFN at a lytic concentration (250 ng/ml), note the intensity of PI staining in the nucleus in this un-enhanced image. Data are representative of 1 of 2 independent experiments; the most in-frame section is shown. Enhancement of select panels was performed with Adobe Photoshop where highlight value for select color channel was in all instances changed from 255 to 100.

To determine physiological relevance, we then asked whether target cells exposed to CTLs develop rapid PS flip-flop. For these studies, ex vivo CD8 T cells from LCMV-immune (day 7) WT, GzmA/B^−/−^ and PFN/GzmA/GzmB^−/−^ mice were added to target cells in the presence and absence of the immunizing peptide, gp 33. PI was added prior to formation of the conjugates to evaluate membrane damage and Ann-V staining of PS flip-flop was measured after the conjugates had been incubated for the times designated. Unlike our studies with isolated human PFN, a discrete subset of targets display low intensity Ann-V staining and subtle PI uptake (Fig. 4d). Presumably the PS flip-flop associated pores that are induced by isolated human PFN are somewhat smaller in size than those induced by the CTLs (see below). As described above for the isolated proteins, adding the combination of PFN and GzmB prevents us from reliably detecting PS flip-flop due to PFN alone (Fig. 4d – right panel). However, the GzmAxB−/− CD8 cells which contain PFN but lack the pro-apoptotic GzmB as well as the non-cytotoxic GzmA [42] rapidly induce low intensity Ann-V reactivity suggesting that ex vivo antigen specific CD8 cells are able to stimulate rapid PS flip-flop in recognized target cells through the action of PFN. Taken together, mouse PFN secreted by antigen specific CD8 cells induces PS flip-flop as well as allows PI to enter target cells where the fluorophore remains minimally excitable in the cytosol compared to the much greater excitation that occurs after the probe binds to nuclear DNA of necrotic cells (see Fig. 5b–c, see below). This pattern is only observable when the probe is added prior to PFN challenge.

### Human PFN can be manipulated to produce low intensity PI cells

Since isolated mouse PFN was unavailable, we explored the possibility that human PFN could also induce simultaneously low intensity PI and Ann-V staining if conditions were modified to increase monomer binding. Reducing the pH of the buffer will increase the density of monomers that bind to target cells. This strategy generates low intensity PI cells [43] and results in the development of low intensity Ann-V+cells (See Figure S1g). However, the relatively harsh incubation at acidic pH and the complex experimental design could introduce systematic errors that might lead to the measurable low intensity Ann-V and PI staining. We, therefore, corroborated this work with a simpler design that involved the incubation of the target cells with PFN in the presence of 0.2 mM Ca. The rationale for this approach was our previous observation that cell associated PFN was more readily detectable by anti-DG9 mAb staining and flow cytometry after incubation of the targets with PFN and 0.2 mM Ca as opposed 1.25 mM Ca. This finding suggested that a greater number of monomers bound to the target cells in the reduced Ca buffer [44], perhaps because fewer were inactivated in the solution phase. Treatment of the Jurkat cells with human PFN in the presence of 0.2 mM Ca produced cells that were both low intensity Ann-V and PI positive (Fig. 5a). To clarify the localization of the PI staining, Jurkat cells that contained low levels of PI by flow cytometry were evaluated by CLSM. The observed low intensity PI staining could be attributed to cytosolic localization of the fluorophore compared to the more intense nuclear fluorescence observed for necrotic cells (Fig. 5b). Imaging of HeLa cells confirmed the cytosolic localization of the low intensity PI (Fig. 5c). These results suggest that structures formed in target cells by mouse and human PFN are similar with the low intensity PI uptake induced by the CD8 cells attributable to a higher net level of PFN binding to the target cells.

## Discussion

How PFN delivers GzmB into cells without causing detectable damage to the cell membrane is a matter of intense interest especially since an understanding of this phenomenon will allow the development of more rational strategies for intra-cellular protein delivery. The evidence presented here suggests that the mechanism involves lipid components of the membrane themselves, as well as PFN monomers inserted into the membrane. For example, single channel conductance measurements show that the physical characteristics of the pore including its size, its stability and its mechanism of formation are affected by the degree of ordering in the membrane lipids [45], [46]. Pore diameters formed by PFN appear to vary depending on membrane composition ranging from homogenous phospholipids of a single headgroup to erythrocyte membrane and the plasma membrane of nucleated cells [1], [5], [8], [9], [11], [12], [47]. Despite stringent efforts to uncover the 20 nm pores, when PFN is applied to target cells at concentrations that contribute to granzyme delivery, even low molecular fluorophores such as PI do not enter the target cells. Instead, we present evidence that PFN reconfigures membrane lipids on live cells that results in unanticipated phospholipid flip-flop. We show by Ann-V and lactadherin staining that PS appears in the outer leaflet of the bilayer, and that this marks cells that are prepared for GzmB delivery. PS flip-flop is not caused by PFN-induced calcium influx and seems to increase when cells are exposed to an oLPC that enhances positive membrane curvature and by cholesterol depletion.

PFN appears to induce flip-flop of PS from the inner to the outer membrane leaflet that does not rely on the induction of intracellular translocases and flippases. The externalization of PS we observe can most easily be explained by the fusion of the inner and outer leaflets of the bilayer and the lipid's migration over the resulting lip, a process that does not occur during the formation of transmembrane proteinaceous channels. A related toroidal structure is known to arise during electroporation [48], [49] as well as during membrane fusion events that occur upon viral cell entry and transfer of intracellular cargo between vesicular compartments [22], [23].

PFN monomers have been reported to clearly oligomerize into a cylindrical pore-forming proteinaceous channel which span the membrane bilayer to form a pore and this mechanism must contribute to certain biological effects mediated by the toxin [5], [45]. Many other proteins are known to form pores using a similar mechanism of oligomerization and insertion. Nevertheless, biophysical data have suggested that bacterial peptides such as melittin [19], [50], toxins including Equinatoxin II [21] and small proteins like Bax [20], all may induce pores consisting of a mixture of incompletely-oligomerized protein and the membrane phospholipids themselves. Whether larger toxins such as bacterial CDCs and the related MACPF domain proteins such as PFN form these structures is highly controversial [51] but must be considered possible given the apparent effects of the membrane lipid components on pore structure, stability and lifetime [45], [52]. Pores formed by such matrices of protein and lipid would have the necessary characteristics to allow PS migration between PM leaflets and would be similar to pore structures induced during membrane fusion [22], [23]. Using a variety of biophysical approaches including electron microscopy [10], atomic force microscopy [53] and X-ray diffraction [20], the existence of these unusual pores in model membranes has been argued to exist. However, even if evidence for their formation *in vitro* is accepted, their appearance in biological membranes has remained unproven, due to their presumed evanescence as shown by transient Ca influx and the lack of technologies capable of imaging complex membranes at the required resolutions.

One of the major concerns in understanding how a membrane is altered by an applied protein is whether its insertion achieves the biological effect observed in vivo. PFN may be added to target cells at concentrations that induce widespread necrosis but such toxic levels neither mirror the conditions necessary for granzyme delivery nor reflect the levels encountered by a target cell after CTL engagement (see Fig. 4). Our data establish for the first time guidelines that allow the identification of physiologically relevant PFN concentrations for cellular and biophysical studies. We hypothesized previously that PFN oligomers might disrupt the plasma membrane allowing granzymes to cross the bilayer in the absence of discrete pores [13]. The phenomena we see evidenced in the movement of PS to the upper leaflet of the membrane would correlate with such an event.

**Figure 6:**
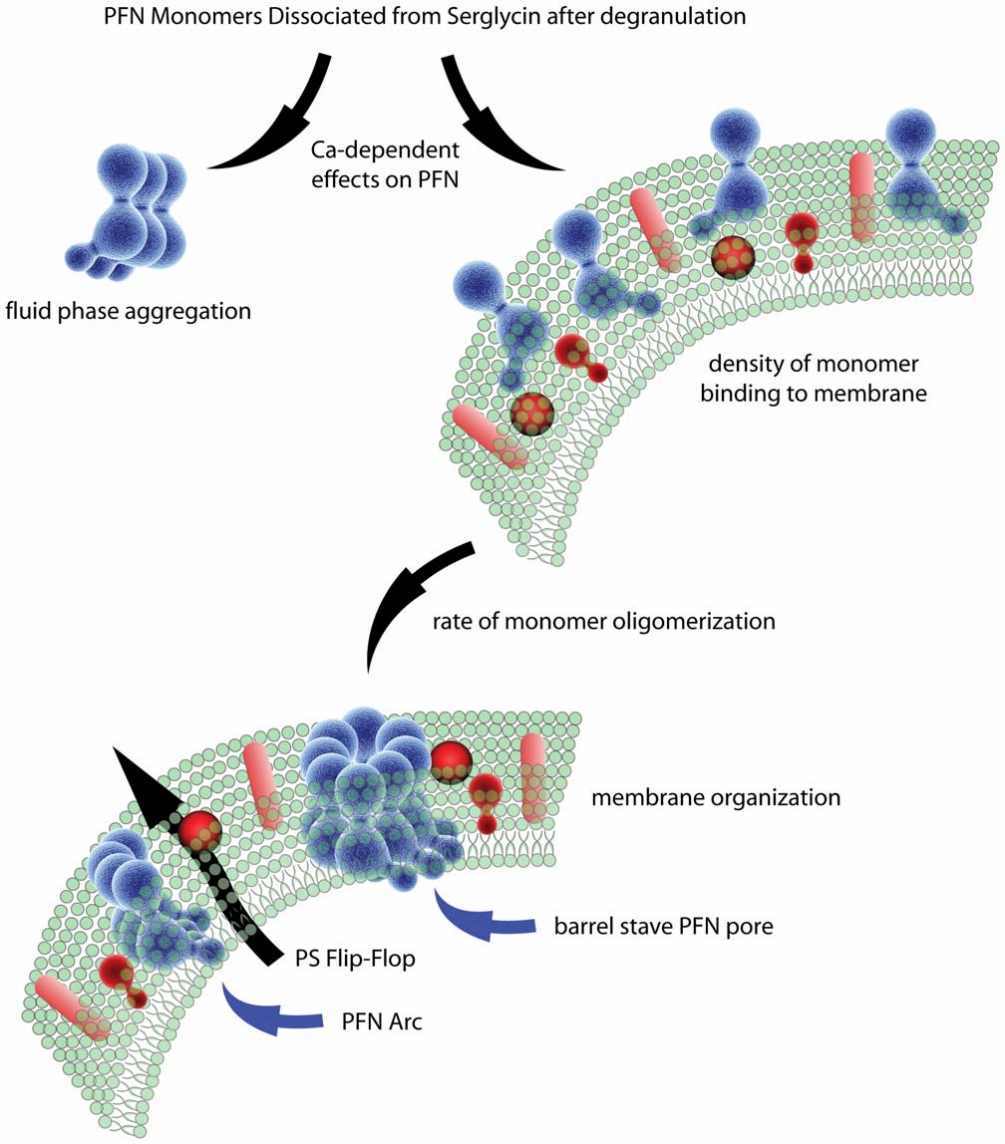
Factors that influence the outcome of PFN interaction with cells. Once secreted from effector cells, PFN monomers are rapidly inactivated by Calcium thereby limiting their availability to interact with the plasma membrane of the target cell. The complexity of the plasma membrane further influences the density of monomer binding and rate of oligomerization. Together these variables may influence the tendency of PFN to form barrel stave pores of varying diameters or, possibly, arcs.

Based on recent structural data, PFN is predicted to oligomerize to a single-sized pore (20 nm) regardless of extracellular conditions or membrane composition that serves as the platform for insertion. However, data reported here and elsewhere suggest that the structures formed by PFN are variable in size [45] depending on at least two factors that influence binding density and oligomerization rates. These include 1) The concentration of Ca ions and inhibitory proteins [24] that PFN encounters and 2) the composition and organization of the membrane that the monomers bind, oligomerize and then insert within. Together these conditions appear to influence the characteristics of pores with cylinders predominating above a concentration threshold while membrane structures that give rise to PS flip-flop are more common when the level of monomers is limiting (Fig. 6). These latter structures may be incomplete ring oligomers, or arcs, that have been observed previously [1], [5].

While PFN is observed to form cylinders and arcs on liposomes and sheep erythrocyte ghosts when evaluated by EM, a systematic description of membrane alterations that might be induced by the toxin has not been undertaken. In this regard, we have learned that PFN induces membrane invaginations in both LUVs and giant unilamellar vesicles (manuscript submitted). Although additional work is necessary to understand how PFN induces such structures, it is intriguing to speculate that PFN arcs may generate positive membrane curvature that partly contributes to the observed invaginations and the counterpart to these morphological alterations is transient membrane fusion and phospholipid flip-flop when PFN is applied to nucleated cells in limiting amounts associated with granzyme delivery. Inasmuch as cylindrical pore structures have not been identified in target cells that would allow passage of the granzymes, the observed flip-flop becomes the first viable pathway that could offer a route for GzmB translocation. However, translocation would depend on the availability of GzmB to interact with membrane phospholipids of the outer leaflet. Since GzmB would preferentially bind the glycosaminoglycan (GAG) side chains of cell surface proteolgycans, a GzmB-phospholipid interaction would require a radical movement of the proteoglycans and their associated Glycosaminoglycan chains outward from the phospholipid binding sites on the leaflet. Such a possibility may occur efficiently in the CTL synapse where major alteration in the topological distribution of integral membrane proteins has been reported.

PFN induced Ca influx is considered to be mechanistically linked to granzyme delivery [17]. In this model, PFN triggers a plasma membrane-repair response that depends on inward movement of Ca ions. The response then drives endocytic uptake of GzmB and its delivery, an outcome inhibited by BAPTA-AM. For these studies, an arbitrary sub-lytic dose of PFN was employed without distinguishing the subset of cells that PFN renders susceptible to GzmB. By adding PI before PFN, we could identify and recognize those cells that succumb to GzmB versus targets that became rapidly necrotic. We were thus surprised to observe that intracellular chelation of PFN-induced Ca influx did not reduce granzyme delivery and apoptosis induction when detected by a highly sensitive probe for caspase-3/-7 activation. This issue notwithstanding, Ca influx and PS flip-flop appear to represent entirely separate biological processes where Ca influx indeed may serve to protect cells as the intracellular levels of Ca rise with increasing deposition of cylindrical pores.

PFN-induced phospholipid flip-flop appears to correlate highly with target cells that undergo GzmB-induced apoptosis, but PS translocation may serve other functions. PFN is considered to act in host defense primarily as the delivery agent for the granzymes. However, PFN has been shown to be both necessary and sufficient in the absence of the granzyme A and B to eliminate tumor burden [54]. PFN alone would be predicted to act by inducing tumor cell necrosis, which is viewed as deleterious to the host by inducing autoimmune responsiveness. Perhaps, PFN-induced phospholipid flip-flop is instead sufficient to stimulate phagocytic clearance of tumor cells though mechanisms described for apoptotic cells.

In summary, the observation that a 5 min pulse of PFN and GzmB is sufficient for apoptosis suggests that the granzyme may simply translocate through structures that reflect PS flip-flop at the plasma membrane. The validity of these concepts await our ability to image target cells treated with the physiologically relevant concentrations of the pore forming protein, to determine whether mutations that alter the oligomerization of membrane bound PFN encourages formation of the proposed arc-like structures, and to learn whether GzmB exploits these structures to translocate to the cytosol.

## Materials and Methods

### Ethics Statement

All experimental work involving mice were performed according to FELASA guidelines under the supervision and approval of Comité Ético para la Experimentación Animal (Ethics Committee for Animal Experimentation) from CITA (Agrifood Research and Technology Centre from Aragon), Approval # CEEA-01, 27/01/2011, N° 2011/01.

### Cell lines and reagents

Jurkat and HeLa cells were purchased from ATCC and cultured in RPMI – 10% fetal bovine serum (FBS) media. All reagents and tissue culture supplies were purchased from either Sigma-Aldrich (St. Louis, MO) or Invitrogen (Carlsbad, CA). Granzyme B (GzmB) and human PFN (PFN) were isolated as described [16], [55]. PFN was then stabilized to tolerate freeze thaw cycles by the addition of 1% fatty acid-free BSA. Eqt II was isolated as described [21] and Streptolysin O (SLO) was a kind gift from S. Bhakdi. Oleoyl Lysophosphatidylcholine (18∶1 Lyso PC; 1-oleoly-2-hydroxy-sn-glycero-3-phosphocholine) was purchased from Avanti Polar Lipids (Alabaster, AL). Sheep red blood cells (SRBC) were purchased from Colorado Serum (Denver, CO). Polyethylene glycol (PEG) polymers of varying Mr (2,000 to 10,000 daltons) and Propidium Iodide (PI) were from Sigma-Aldrich (St. Louis, MO). The terminal deoxynucleotidyl transferase dUTP nick end labeling (TUNEL) and Annexin-V-FITC kits were from BD Biosciences (San Jose, CA). MitoTracker red (CMXRos), Fluo-3, Fluo-4, Fura Red, the intracellular Calcium chelator, BAPTA-AM, and the Cell Event Caspase 3/7 detection reagent were from Invitrogen (Carlsbad, CA). Bovine Lactadherin conjugated to FITC was from Hematological Technologies (Essex Junction, VT).

### Production of the lethal hit

Target cells were suspended at 1×10^7^ per ml in the high Ca buffer (HCB) that consisted of 150 mM NaCl, 20 mM Hepes and 2.5 mM Ca (pH 7.4). PFN was diluted in a no calcium buffer (NCB) that consisted of 150 mM NaCl, 20 mM Hepes, and 1% BSA (pH 7.4). PFN was added to microwells of a 96 well plate. GzmB, at desired concentration, was then added (50 µl) followed by target cells (50 µl) and incubated for 5 min at 37°C. PI was present at final concentration of 10 µg/ml throughout. The cells were then washed twice with PBS containing 5 mM EGTA followed by single wash with PBS and resuspended in 1 ml of RPMI, 10% FBS. The cells were separated into two aliquots where the first was exposed to MitoTracker red CMXRos (50 nM) while the second received additional PI (10 µg/ml). After 45 min, the cells were analyzed by flow cytometry.

### Cell death assays

#### Mitochondrial depolarization assay

Mitochondrial potential loss using CMXRos was measured as reported [56]. Cell viability was measured using PI (10 µg/ml) and flow cytometry.

#### TUNEL assay

Target cells were pulsed with PFN in presence of GzmB for 5 min as described above, washed with EGTA and incubated for 90 min. Cells were then fixed and death measured by TUNEL [16].

#### Activation of Caspase3/7

Target cells were treated with PFN and GzmB as described above in presence of the Cell Event Caspase 3/7 detection reagent (5 µM, Invitrogen, Carlsbad, CA), and PI for 5 min (pulse) or continuously for 1 hour incubation at 37°C. PI was present at final concentration of 10 µg/ml throughout. In case of a pulse, after an EGTA wash, the cells were resuspended in buffer containing PI and Cell Event Caspase 3/7 reagent, allowed to incubate for 55 min at 37°C and analyzed by flow cytometry.

### Calcium influx in PFN treated targets

Jurkat cells preloaded with Fluo-3 and Fura Red (1 µM each) were treated with PFN in presence of PI (10 µg/ml) and flow cytometry data acquired in real-time (up to 360 seconds). The final extracellular calcium concentration was 1.25 mM. Cells in the PI negative gate (Fluorescence intensity <10) were analyzed for Fluo-3 and Fura Red fluorescence and data expressed as the ratio of MFI for Fluo-3 over Fura Red. Ionomycin treated cells served as positive controls.

In experiments utilizing the intracellular calcium chelator BAPTA-AM, calcium flux was measured using Fluo-4. Cells were loaded with 100 µM BAPTA-AM and Fluo-4 (5 µM) for 40 min at room temperature in the dark. Cells were then washed and treated with PFN in presence of PI (10 µg/ml) and changes in Fluo-4 fluorescence measured in real time over 360 sec. Cells in the PI negative gate were analyzed for Fluo-4 fluorescence and data was expressed as MFI for Fluo-4 in treated samples minus MFI in control sample (background subtracted).

The effect of Calcium chelation on PFN mediated GzmB delivery was studied using the Caspase 3/7 reagent. Here cells were first loaded with DMSO (vehicle control) or BAPTA as described above, followed by washes and then treatment with GzmB and PFN for 1 hour at 37°C in presence of PI and Cell Event Caspase 3/7 detection reagent (5 µM).

### PFN induced PS flip-flop in targets measured by Annexin V binding

Cells were treated with designated concentrations of PFN for 5 to 15 min in presence of PI (10 µg/ml) at a final extracellular calcium concentration of 1.25 mM. Cells were then washed and stained with Ann-V-FITC per the manufacturer's instructions. Data were acquired as Ann-V-FITC versus PI density plots. Equinatoxin II (Eqt II) and Streptolysin O (SLO) were used in select experiments as controls to monitor formation of proteo-lipid and barrel stave pores respectively.

### PFN induced PS flip-flop in targets undergoing intracellular calcium chelation

To test whether PFN induced PS flip-flop was dependant on calcium activated PS translocase activity, intracellular calcium chelation was performed using BAPTA-AM. Here cells were loaded with BAPTA-AM (100 and 50 µM) or DMSO (vehicle control) in RPMI, 1% BSA for 40 min at RT. Cells were then washed and treated with either human or rat PFN for 15 min at 37°C with extracellular calcium concentration of 1.25 mM. Ann-V-FITC staining was then performed. PI (10 µg/ml) was present through out the assay.

### PFN induced PS flip-flop in targets undergoing GzmB induced apoptosis

To distinguish the pattern of Ann-V-FITC staining in presence of PFN alone versus PFN-mediated GzmB delivery (apoptosis), target cells were pulsed with PFN in the presence or absence of GzmB (1 µg/ml) for 15 min at 37°C. Ann-V-FITC staining was performed immediately with PI (10 µg/ml) present throughout the assay. To ascertain the pattern of Ann-V-FITC staining in a typical apoptosis assay, cells were treated with PFN in the presence or absence of GzmB (1 µg/ml) for 120 min at 37°C followed by Ann-V staining.

### PFN induced PS flip-flop measured by Lactadherin binding

Cells were treated with human PFN for 15 min in presence of 10 µg/ml PI at a final extracellular calcium concentration of 1.25 mM. Cells were then washed and stained with lactadherin-FITC per manufacturer's instructions. PI was present throughout the assay. Data was acquired as lactadherin-FITC versus PI density plot.

### Effect of oleoyl Lysophosphatidylcholine on PFN induced PS flip-flop

To test whether the positive curvature lipid, oleoyl Lysophosphatidylcholine (oLPC) could augment PFN induced PS flip-flop, cells were incubated with oLPC (8 and 4 µM) for 15 min at 37°C in absence of BSA. Cells were then washed once in 1.25 mM CaCl_2_ containing 150 mM NaCl, 20 mM Hepes, pH 7.4 and resuspended in the same buffer supplemented with 0.125% BSA. Cells were incubated with PFN for 15 min at 37°C, washed and stained with Ann-V-FITC. PI (10 µg/ml) was present throughout the assay.

To test the effect of oleoyl Lysophosphatidylcholine on Eqt II induced PS flip flop, cells were incubated concomitantly with oLPC (4 µM) and Eqt II at the indicated concentrations for 15 min at 37°C in absence of BSA. Cells were then washed once in 1.25 mM CaCl_2_ containing 150 mM NaCl, 20 mM Hepes, pH 7.4, 0.5% BSA and stained with Ann-V-FITC. PI (10 µg/ml) was present throughout the assay.

### Effect of Methyl-β-cyclodextrin (MBCD) on PFN and Eqt II induced PS flip-flop

To test the effect of cholesterol depletion on PFN and Eqt II induced PS flip-flop, cells were washed twice in 2.5 mM CaCl_2_ containing 150 mM NaCl, 20 mM Hepes, pH 7.4 buffer. They were resuspended in the same buffer and treated with 0 or 1.5 mM MBCD for 1 hr at 37°C. After 1 hr, the cells were plated in wells containing equal volume of PFN or Eqt II diluted in 150 mM NaCl, 20 mM Hepes, pH 7.4 buffer containing 0.5% BSA. After a 15 min treatment at 37°C, cells were washed and stained with Ann-V-FITC. PI (10 µg/ml) was present throughout the assay.

### PFN induced PS flip-flop in targets under low Calcium

Cells were treated with designated concentrations of PFN for 15 min in presence of PI (10 µg/ml) at a final extracellular calcium concentration of 0.2 mM. Cells were then washed with 0.2 mM calcium containing 150 mM NaCl, 20 mM Hepes, pH 7.4 buffer and stained with Ann-V-FITC in presence of 1.25 mM calcium containing 150 mM NaCl, 20 mM Hepes, pH 7.4 buffer, 0.5% BSA. Data were acquired as Ann-V-FITC versus PI density plots.

### Imaging of targets that exhibit low intensity PI staining after PFN treatment

Jurkat cells were incubated with PFN in the presence of PI (10 µg/ml) for 30 min at 37°C in 1.25 mM Calcium containing Hepes-NaCl buffer, pH 7.4. Cells were washed and an aliquot analyzed by flow cytometry. PFN concentrations yielding a substantial PI low population (fluorescence intensity ranging between 10 and 600) were selected for imaging. Cells were layered on to poly-L-lysine coated slides, allowed to adhere and imaged on a Leica Confocal Laser Scanning Microscope System with a DMIRE2 inverted microscope (Leica Microsystems, Exton PA). Twelve-bit fluorescence images of the fluorophores were acquired with 100 X oil-immersion objectives (N.A. 1.25 and 1.4 respectively) using the Leica Confocal software. In experiments with HeLa, cells were plated on coverslips overnight. Cells were then washed and treated with PFN in presence of FM1-43 (5 µM) and PI.

### Preparation and use of ex vivo CTLs

Mice (WT, gzmAxB^−/−^, and pfnxgzmAxB^−/−^) were inoculated with 10^5^ pfu LCMV followed by splenectomy 8 days later. CD8+ cells were positively selected (MACS), stained with Cell tracker green (1 µM) and added to EL4 cells in the presence and absence of the immunizing peptide, gp33, at an effector∶ target (E∶T) ratio of 2∶1 [57]. PI (10 µg/ml) was added at onset with PI and Ann-V reactivity of targets determined after 10, 20 and 120 min. Under this immunizing protocol, similar numbers of LCMV-specific CTLs (analyzed by tetramer staining) were recovered from the three mouse strains.

Methodology used to generate supporting information is included under Methods S1.

## Supporting Information

Figure S1 **a:** *Calcium inactivates PFN in fluid phase.* Human PFN was incubated in 1.25 mM Ca buffer – 0.5% BSA buffer at 37°C for the indicated times and then incubated with cells for an additional 20 min in presence of PI (10 µg/ml). Samples were then run on the cytometer to enumerate the PI Hi cells. **b:** *Fluid phase PFN structures observed by cryo-EM*: Electron micrograph of PFN oligomers in fluid phase in presence of Calcium. PFN was incubated with 2 mM Ca and the resulting oligomers stained with uranyl acetate. Images were grouped by multivariate statistical analysis using the program IMAGIC and the classes of similar images averaged to produce these class sums. The numbers given indicate the size of each class (number of individual images it comprises) and the associated percentages. Scale bar, 20 nm. **c:** *Five min pulse with PFN and GzmB is sufficient to induce Caspase 3/7 activation.* Jurkat cells were incubated with PFN and GzmB (1 µg/ml) at the indicated concentrations for 1 hr in a continuous incubation (left) or pulsed with GzmB and PFN for 5 min (right). For the pulse set, the reaction was stopped by an EGTA wash step and cells incubated for another 55 min in presence of PI and Cell Event Caspase3/7 reagent after which they were analysed by flow cytometry (data for one of two experiments). **d:** *Sizing PFN pores in SRBCs using the PEG osmotic protection assay.* SRBCs were exposed to increasing concentrations of human PFN in the presence and absence of PEGs ranging from 2,000 through 6,000 dalton. Hemoglobin release was determined as described in methods; data represents one of two experiments. Background Hemoglobin release ranged from 8 to 9.1%. **e:** *Membrane repair after SLO requires extracellular Calcium.* Jurkat cells were treated with the indicated concentrations of SLO for 15 min at 37°C in presence or absence of Calcium and acquired on the cytometer. Data for one representative experiments of two is shown. **f:** *Typical pattern of Ann-V and PI during GzmB induced apoptosis.* To compare the pattern of Ann-V-FITC staining produced by PFN versus PFN plus GzmB, cells were treated with PFN in the presence and absence of GzmB (1 µg/ml) for 120 min at 37°C followed by Ann-V-FITC staining (data for one of two experiments). **g:** *Target cells coated with human PFN at pH 6.0 develop both low intensity PI and Ann-V reactivity.* Jurkat cells were treated with human PFN (pH 6) in the presence of calcium and Na Azide at 4°C, washed (pH 7.4) at 4°C and then incubated with 1.25 mM calcium at 37°C for 15 min. Thereafter, cells were stained with Ann-V-FITC as described (one of two experiments).
(TIF)

Methods S1 **Methodology used to generate supporting information.**
(DOC)
