# Supplementary figures and images for: Perforin Rapidly Induces Plasma Membrane Phospholipid Flip-Flop
Source: PLoS One. 2011 Sep 12;6(9):e24286. doi: 10.1371/journal.pone.0024286 (PMC3171411; doi:10.1371/journal.pone.0024286)

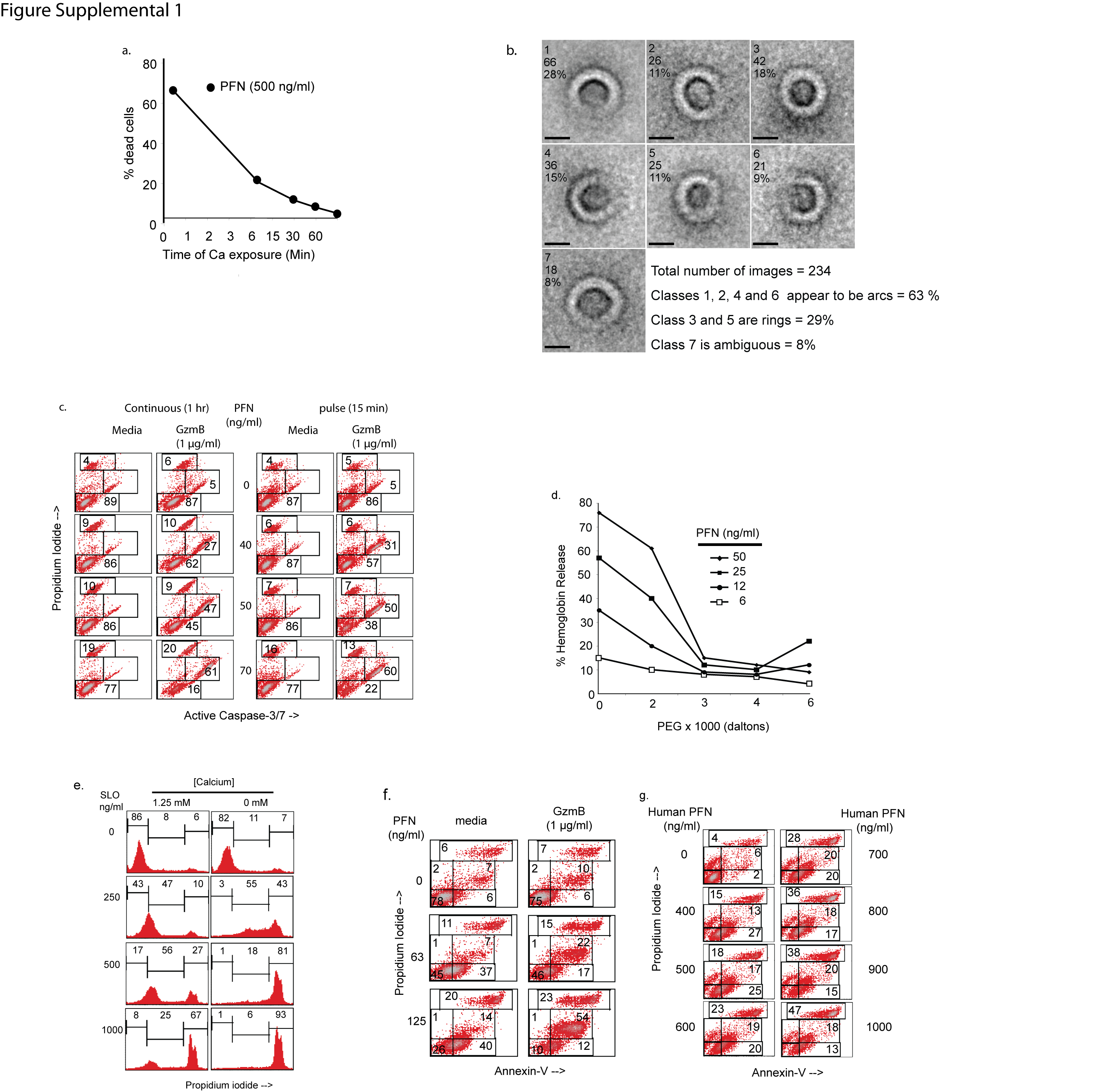

Supplement: Figure S1 — a: Calcium inactivates PFN in fluid phase. Human PFN was incubated in 1.25 mM Ca buffer – 0.5% BSA buffer at 37°C for the indicated times and then incubated with cells for an additional 20 min in presence of PI (10 µg/ml). Samples were then run on the cytometer to enumerate the PI Hi cells. b: Fluid phase PFN structures observed by cryo-EM: Electron micrograph of PFN oligomers in fluid phase in presence of Calcium. PFN was incubated with 2 mM Ca and the resulting oligomers stained with uranyl acetate. Images were grouped by multivariate statistical analysis using the program IMAGIC and the classes of similar images averaged to produce these class sums. The numbers given indicate the size of each class (number of individual images it comprises) and the associated percentages. Scale bar, 20 nm. c: Five min pulse with PFN and GzmB is sufficient to induce Caspase 3/7 activation. Jurkat cells were incubated with PFN and GzmB (1 µg/ml) at the indicated concentrations for 1 hr in a continuous incubation (left) or pulsed with GzmB and PFN for 5 min (right). For the pulse set, the reaction was stopped by an EGTA wash step and cells incubated for another 55 min in presence of PI and Cell Event Caspase3/7 reagent after which they were analysed by flow cytometry (data for one of two experiments). d: Sizing PFN pores in SRBCs using the PEG osmotic protection assay. SRBCs were exposed to increasing concentrations of human PFN in the presence and absence of PEGs ranging from 2,000 through 6,000 dalton. Hemoglobin release was determined as described in methods; data represents one of two experiments. Background Hemoglobin release ranged from 8 to 9.1%. e: Membrane repair after SLO requires extracellular Calcium. Jurkat cells were treated with the indicated concentrations of SLO for 15 min at 37°C in presence or absence of Calcium and acquired on the cytometer. Data for one representative experiments of two is shown. f: Typical pattern of Ann-V and PI during GzmB induced apoptosis. [file pone.0024286.s001.tif]
