# Supplementary material for: Perforin Rapidly Induces Plasma Membrane Phospholipid Flip-Flop
Source: PLoS One. 2011 Sep 12;6(9):e24286. doi: 10.1371/journal.pone.0024286 (PMC3171411; doi:10.1371/journal.pone.0024286)
Supplement: Methods S1 — Methodology used to generate supporting information. (DOC) [file pone.0024286.s002.doc]

**Supplemental Methods**:

*Polyethylene glycol protection assay to size PFN pores in sheep RBCs.* SRBC were washed in HCB and resuspended at 0.4% (v/v). PEG and PFN were diluted in NCB. Reaction volumes (200 µl) contained 90 µl PEG (30 mM), 100 µl SRBC (0.2%), and 10 µl PFN (as indicated) in 96-well U-bottom plate. The plate was incubated at 37°C for 20 min, centrifuged at 4°C to pellet SRBC and 150 µl of the supernatant was transferred into a fresh 96-well flat-bottom plate with abs_405_ measured in a microplate reader. The per cent hemoglobin release equaled [(A_405_ (sample) - A_405_ (blank))/(A_405_ (detergent)- A_405_ (blank))] x 100%, where blank contained all components except PEG and the detergent reaction was the same as the blank containing 0.2% Triton X-100 (Sigma).

*Production of the lethal hit – Activation of Caspase3/7:* Target cells were suspended at 1 x 10^7^ per ml in the high Ca buffer (HCB) that consisted of 150 mM NaCl, 20 mM Hepes and 2.5 mM Ca (pH 7.4). PFN was diluted in a no calcium buffer (NCB) that consisted of 150 mM NaCl, 20 mM Hepes, and 1% BSA (pH 7.4). PFN was added to microwells of a 96 well plate. GzmB (1 µg/ml, 50 µl) was then added along with the Cell Event Caspase 3/7 detection reagent (5 µM, Invitrogen, Carlsbad, CA), followed by target cells (50 µl) and incubated for 5 min at 37°C. PI was present at final concentration of 10 µg/ml throughout. The cells were then washed once with PBS containing 5 mM EGTA followed by single wash with 150 mM NaCl, 20 mM Hepes, 1.25 mM Ca (pH 7.4) – 0.5% BSA and resuspended in the same buffer containing PI and Cell Event Caspase 3/7 reagent. After a 55 min incubation at 37°C, cells were analyzed by flow cytometry.

*Effect of extra-cellular calcium on PI entry induced by SLO:* The goal was to delineate the influence of Ca-mediated membrane repair on PI entry induced by SLO. The toxin was incubated with the target cells for 15 min in presence or absence of Ca (1.25 mM). PI (10 µg/ml) was present through the length of the assay.

*Cryo-EM:* PFN at 100 µg/ml was incubated for 10 minutes with 2mM Ca at room temperature. It was then applied to a glow-dicharged carbon-coated EM grid and stained with 3 x 2ul unbuffered 2% w/v uranyl acetate, wicking away the stain after each application and then allowing the grid to air dry. The samples were flash-frozen and imaged with a FEI F30 FEG cryo-electron microscope (Europe NanoPort, Netherlands) operating at liquid nitrogen temperatures.
